# Mapping and Preserving the Visuospatial Network by repetitive nTMS and DTI Tractography in Patients With Right Parietal Lobe Tumors

**DOI:** 10.3389/fonc.2021.677172

**Published:** 2021-06-25

**Authors:** Giovanni Raffa, Maria Catena Quattropani, Giuseppina Marzano, Antonello Curcio, Vincenzo Rizzo, Gabriella Sebestyén, Viktória Tamás, András Büki, Antonino Germanò

**Affiliations:** ^1^ Division of Neurosurgery, BIOMORF Department, University of Messina, Messina, Italy; ^2^ Department of Clinical and Experimental Medicine, University of Messina, Messina, Italy; ^3^ Division of Neurology, Department of Clinical and Experimental Medicine, University of Messina, Messina, Italy; ^4^ Department of Neurosurgery, Medical School, University of Pécs, Pécs, Hungary

**Keywords:** brain tumors, diffusion tensor imaging tractography, navigated transcranial magnetic stimulation (nTMS), superior longitudinal fasciculus (SLF), visuospatial abilities, visuospatial network, parietal lobe, Hooper visual organization test

## Abstract

**Introduction:**

The goal of brain tumor surgery is the maximal resection of neoplastic tissue, while preserving the adjacent functional brain tissues. The identification of functional networks involved in complex brain functions, including visuospatial abilities (VSAs), is usually difficult. We report our preliminary experience using a preoperative planning based on the combination of navigated transcranial magnetic stimulation (nTMS) and DTI tractography to provide the preoperative 3D reconstruction of the visuospatial (VS) cortico-subcortical network in patients with right parietal lobe tumors.

**Material and Methods:**

Patients affected by right parietal lobe tumors underwent mapping of both hemispheres using an nTMS-implemented version of the Hooper Visual Organization Test (HVOT) to identify cortical areas involved in the VS network. DTI tractography was used to compute the subcortical component of the network, consisting of the three branches of the superior longitudinal fasciculus (SLF). The 3D reconstruction of the VS network was used to plan and guide the safest surgical approach to resect the tumor and avoid damage to the network. We retrospectively analyzed the cortical distribution of nTMS-induced errors, and assessed the impact of the planning on surgery by analyzing the extent of tumor resection (EOR) and the occurrence of postoperative VSAs deficits in comparison with a matched historical control group of patients operated without using the nTMS-based preoperative reconstruction of the VS network.

**Results:**

Twenty patients were enrolled in the study (Group A). The error rate (ER) induced by nTMS was higher in the right vs. the left hemisphere (p=0.02). In the right hemisphere, the ER was higher in the anterior supramarginal gyrus (aSMG) (1.7%), angular gyrus (1.4%) superior parietal lobule (SPL) (1.3%), and dorsal lateral occipital gyrus (dLoG) (1.2%). The reconstruction of the cortico-subcortical VS network was successfully used to plan and guide tumor resection. A gross total resection (GTR) was achieved in 85% of cases. After surgery no new VSAs deficits were observed and a slightly significant improvement of the HVOT score (p=0.02) was documented. The historical control group (Group B) included 20 patients matched for main clinical characteristics with patients in Group A, operated without the support of the nTMS-based planning. A GTR was achieved in 90% of cases, but the postoperative HVOT score resulted to be worsened as compared to the preoperative period (p=0.03). The comparison between groups showed a significantly improved postoperative HVOT score in Group A vs. Group B (p=0.03).

**Conclusions:**

The nTMS-implemented HVOT is a feasible approach to map cortical areas involved in VSAs. It can be combined with DTI tractography, thus providing a reconstruction of the VS network that could guide neurosurgeons to preserve the VS network during tumor resection, thus reducing the occurrence of postoperative VSAs deficits as compared to standard asleep surgery.

## Introduction

The modern goal of brain tumor surgery is to achieve the so-called “maximal safe resection”, consisting in the maximal resection of neoplastic tissue while respecting adjacent eloquent tissue to preserve brain functions (1–3). Traditionally, motor and language functions can be confidentially assessed by neurosurgeons both pre- and postoperatively, therefore great attention is usually paid to the preservation of cortico-subcortical networks involved in these functions before and during brain tumor surgery (3–7). Conversely, complex cognitive functions, including visuospatial abilities (VSAs), usually require a specific neuropsychological expertise for their assessment, and therefore are usually undervalued by neurosurgeons when facing with surgery of brain neoplasms (8). Nevertheless, many cognitive functions are equally important than motor and language functions for an optimal quality of life, and should be preserved during brain tumor surgery to ensure, as much as possible, a normal postoperative familial, social, and even professional life to patients (8). Nevertheless, the neuroanatomical correlates of these brain functions are not well known or understood in all cases, and therefore the preservation of the involved cortico-subcortical networks cannot be easily achieved during brain tumor surgery.

Among these functions, VSAs deserves a great consideration, since it consists of a heterogeneous group of cognitive processes involved in the visual interaction with the environment and objects, essential for visual perception and finalistic behavior (9). VSAs impairment can result in a variety of neurological manifestations, including hemispatial neglect, visuospatial agnosia, etc. that severely affect the everyday life of patients, their quality of life (9, 10), and potentially their Karnofsky performance status, thus potentially also influencing their eligibility to adjuvant oncological care. The neuroanatomical correlate of VSAs has been identified mainly in right hemisphere, consisting in a fronto-parietal network mainly represented by the posterior parietal cortex and its connections with the prefrontal cortex by the superior longitudinal fasciculus (SLF), and in particular its three branches, namely the SLF-I, SLF-II, and SLF-III (11–16). Such a hemispheric asymmetry results in the fact that the right hemisphere controls attentional orienting in both left and right hemispaces, while the left hemisphere controls the direction of attention only in the right hemispace (17–21).

The visuospatial (VS) network can be successfully indentified and preserved during brain tumor surgery by expert neurosurgeons in collaboration with neuropsychologists during awake surgery (8, 10). Nevertheless, not all patients are eligible to undergo awake surgery (22, 23), and unfortunately to date many neurosurgical centers does not possess the expertise and resources to perform intraoperative brain mapping of the VS network (8). The final result is that, still nowadays, the attention paid to the VS network during brain tumor surgery is poor, and the occurrence of postoperative VSAs defictis is underestimated (8).

An alternative to intraoperative mapping is represented by advanced functional and structural imaging. Functional MRI (fMRI) can be used to identify the fronto-parietal cortical areas of the VS network (24). Nevertheless, fMRI has the limitation to show wide activation regions that reflect all the cognitive processes involved in the execution of a specific task, but not necessarily essential for it (25–28). Moreover, the phenomenon of dissociation between neural activation and BOLD signal has been widely reported and represents a serious potential risk for misinterpretation of fMRI results (29), even when using visual tasks in experimental models (30). Therefore, in the past years, several studies have tried to map cortical areas of the VS network by using different technologies, including repetitive navigated transcranial magnetic stimulation (nTMS) (31–33). nTMS has a higher spatial resolution (few millimeters) as compared to fMRI (34–36), and, unlike fMRI that relies on a positive-activation model, repetitive nTMS is based on a virtual-lesion model, consisting in a transient disruption of neuronal activity through a repetitive stimulation during the execution of a specific task (37, 38). Preliminary reports demonstrated repetitive nTMS is a feasible technique to successfully map cortical areas of the VS network in healthy subjects (31–33). On the other hand, diffusion tensor imaging (DTI) tractography can be successfully used to compute the three major branches of the SLF (SLF-I, SLF-II, SLF-III), and to visualize their connections to the right parietal and frontal lobe, thus enabling the identification of subcortical white matter fibers of the VS network (39).

Despite the combination of nTMS and DTI tractography can help neurosurgeons to identify and visualize the cortico-subcortical components of the VS network, to our knowledge no studies have ever analyzed the potential benefit of using such information to identify and preserve the VS network during brain tumor surgery, so far.

Here we report our preliminary experience using a preoperative planning based on nTMS cortical mapping and DTI tractography for the 3D reconstruction and visualization of the VS network. We also analyze how the use of this planning could help neurosurgeons in preserving the network during surgery of brain tumors in the right parietal lobe, as well as in reducing the occurrence of new postoperative visuospatial deficits. Finally, we compared findings achieved in patients treated using our new protocol for preoperative mapping of the VS network with findings observed in a matched historical control group of patients operated using standard asleep surgery without the help of any preoperative functional mapping and planning.

## Materials and Methods

### Study Design

We retrospectively collected clinical, neurological, neuropsychological, and neuroradiological data of all patients admitted at the Department of Neurosurgery of the University of Messina, Italy, between January 2019 and December 2020 harboring a brain tumors located mainly in the right parietal lobe, and submitted to preoperative nTMS mapping of VS cortical areas and DTI tractography of the three SLF branches (SLF-I, SLF-II, SLF-III) to plan and guide tumor resection (Group A). Inclusion criteria were: age ≥ 18 years old, native Italian-language speakers, brain tumors mainly located in the right parietal lobe and therefore suspected to involve the VS network. Exclusion criteria were: age < 18 years old, bilingual speaking, the presence of any contraindication to undergo MRI and/or nTMS mapping (e.g., subjects harboring pacemakers, cochlear implants, non-MRI-compatible prosthesis, severe epilepsy). The information provided by nTMS cortical mapping and DTI tractography enabled the reconstruction of the VS network that was used to plan and guide the maximal tumor resection as well as to preserve the VS cortical and subcortical structures.

We also collected data from a historical control group including 20 patients affected by brain tumors mainly located in the right parietal lobe and operated at the same Neurosurgical Center in the period between January 2016 and December 2020 using a standard asleep microneurosurgical treatment without the use of any preoperative mapping and reconstruction of the VS network (Group B). Patients in Group B were matched for main clinical characteristics with patients in Group A (**Table 1**).

**Table 1:** Salient demographic and clinical characteristics of patients in Group A and B, as well as nTMS mapping features of patients in Group A.

N°	Sex	Age	Handedness	Histology	Location	RH RMT (μV)	LH RMT (μV)	Eloquence for VS network	EOR	HVOT T score (pre-OP)	HVOT T score (post-OP 1 month)	KPS score (pre-OP)	KPS score (post-OP 1 month)	LBT score (pre-OP)	LBT score (post-OP)
**nTMS Group (Group A)**															
#1	F	73	Right	Glioblastoma WHO IV	Fronto-parietal, Right	41	35	N	GTR	68	71	90	90	8	7
#2	M	56	Right	Glioblastoma WHO IV	Parietal, Right	35	38	N	GTR	81	80	80	80	6	7
#3	M	54	Right	Glioblastoma WHO IV	Temporo-parietal, Right	32	36	N	GTR	66	64	80	90	7	8
#4	M	67	Right	Glioblastoma WHO IV	Fronto-parietal, Right	36	29	Y	STR	77	78	70	70	7	7
#5	F	78	Right	Glioblastoma WHO IV	Fronto-parietal, Right	39	38	N	GTR	58	54	80	80	8	9
#6	F	70	Right	Glioblastoma WHO IV	Temporo-parietal, Right	59	41	N	GTR	77	70	90	90	7	7
#7	F	73	Right	Glioblastoma WHO IV	Parieto-occipital, Right	32	31	N	GTR	75	71	80	80	7	7
#8	M	46	Right	Metastases from Lung Cancer	Parietal, Right	30	28	Y	GTR	80	80	80	80	6	6
#9	M	55	Right	Diffuse Astrocytoma WHO II	Fronto-temporo-parietal, right	34	32	N	GTR	71	70	90	90	6	7
#10	M	77	Right	Glioblastoma WHO IV	Parieto-temporo-occipital, right	35	31	N	GTR	76	77	90	80	6	6
#11	M	48	Right	Glioblastoma WHO IV	Parietal, Right	35	34	Y	GTR	80	78	80	90	7	7
#12	F	44	Right	Glioblastoma WHO IV	Parietal, Right	32	32	N	GTR	54	56	90	90	9	9
#13	F	44	Right	Glioblastoma WHO IV	Parietal, Right	38	39	N	GTR	62	60	90	80	9	9
#14	F	53	Right	Glioblastoma WHO IV	Parietal, Right	40	41	N	GTR	74	72	90	90	8	9
#15	F	41	Right	Diffuse Astrocytoma WHO II	Parietal, Right	39	39	Y	GTR	80	80	80	80	7	7
#16	M	66	Right	Glioblastoma WHO IV	Fronto-parietal, Right	34	36	Y	STR	60	60	90	90	7	8
#17	F	65	Right	Glioblastoma WHO IV	Fronto-parietal, Right	41	39	Y	GTR	64	61	80	90	7	7
#18	M	66	Right	Glioblastoma WHO IV	Parietal, Right	31	32	N	GTR	76	74	80	60	7	7
#19	M	25	Right	Diffuse Astrocytoma WHO II	Fronto-parietal, Right	35	36	Y	STR	70	70	90	90	7	7
#20	F	66	Right	Glioblastoma WHO IV	Fronto-parietal, Right	39	39	Y	GTR	68	66	70	80	7	7
**Historical Matched Control Group (Group B)**															
#1	M	57	Right	Glioblastoma WHO IV	Temporo-parietal, Right	/	/	/	GTR	70	72	80	90	7	7
#2	M	52	Right	Glioblastoma WHO IV	Parieto-occipital, Right	/	/	/	GTR	78	76	80	80	7	7
#3	F	68	Right	Glioblastoma WHO IV	Parietal, Right	/	/	/	GTR	68	74	90	90	8	7
#4	F	70	Right	Glioblastoma WHO IV	Fronto-parietal, Right	/	/	/	STR	75	80	70	70	6	6
#5	F	50	Right	Metastases from Breast Cancer	Parietal, Right	/	/	/	GTR	70	72	90	90	6	6
#6	M	71	Right	Glioblastoma WHO IV	Parietal, Right	/	/	/	GTR	67	67	90	90	7	7
#7	F	72	Right	Glioblastoma WHO IV	Fronto-parietal, Right	/	/	/	GTR	72	74	80	80	7	7
#8	M	41	Right	Diffuse Astrocytoma WHO II	Fronto-parietal, Right	/	/	/	STR	84	85	70	60	6	6
#9	M	35	Right	Diffuse Astrocytoma WHO II	Temporo-parietal, Right	/	/	/	GTR	80	80	90	90	6	6
#10	M	50	Right	Glioblastoma WHO IV	Parietal, Right	/	/	/	GTR	70	72	80	90	7	6
#11	M	49	Right	Diffuse Astrocytoma WHO II	Temporo-parietal, Right	/	/	/	GTR	78	74	80	80	7	7
#12	F	50	Right	Glioblastoma WHO IV	Fronto-parietal, Right	/	/	/	GTR	70	70	90	70	7	6
#13	F	55	Right	Glioblastoma WHO IV	Fronto-parietal, Right	/	/	/	GTR	71	72	80	80	7	7
#14	M	75	Right	Metastases from Lung Cancer	Parietal, Right	/	/	/	GTR	68	70	90	80	8	7
#15	M	58	Right	Anaplastic Astrocytoma WHO III	Temporo-parieto-occipital, Right	/	/	/	GTR	72	75	90	90	7	7
#16	M	60	Right	Glioblastoma WHO IV	Parietal, Right	/	/	/	GTR	65	76	90	80	7	5
#17	M	66	Right	Glioblastoma WHO IV	Fronto-parietal, Right	/	/	/	GTR	60	80	80	70	9	6
#18	F	67	Right	Glioblastoma WHO IV	Fronto-parietal, Right	/	/	/	GTR	72	70	80	90	8	8
#19	F	75	Right	Glioblastoma WHO IV	Parietal, Right	/	/	/	GTR	68	69	90	90	8	8
#20	M	52	Right	Diffuse Astrocytoma WHO II	Temporo-parietal, Right	/	/	/	GTR	74	78	90	90	7	6

RH, Right Hemipsher; RMT, resting motor threshold; HVOT, Hooper visual organization test; KPS, Karnofsky Performance Status; LBT, Line Bisection Task; LH, Left Hemisphere; VS, Visuospatial; WHO, World Health Organization.

"/" means not available.

In Group A, we first analyzed the cortical distribution of errors induced by nTMS mapping during the execution of a specific neuropsychological test investigating VSAs to disclose cortical areas involved in the VS network (i.e., the Hooper Visual Organization Test, HVOT) in the right vs. left hemisphere. We also analyzed the eventual different intra-hemispheric distribution of errors induced by nTMS mapping in each hemisphere.

Finally, we analyzed the impact of the use of the preoperative planning based on the advanced reconstruction of the VS network on tumor extent of resection (EOR) and postoperative preservation of visuospatial abilities. We also compared the EOR, the postoperative visuospatial performance and functional outcome in Group A vs. Group B.

All participants signed a written informed consent for collection and use of clinical data for scientific purposes, according to the IRB at our Institution (Comitato Etico Messina).

### Repetitive nTMS Cortical Mapping of the VS Network in Group A

All participants (Group A and B) underwent brain MRI scan by using a 3 Tesla scanner (Achieva 3T, Philips Medical Systems, The Netherlands). T1-weighted multiplanar reconstruction (MPR) sequences (TR/repetition time=8.1, TE/echo time = 3.7) were acquired after gadolinium i.v. administration, as a part of the routine preoperative diagnostic assessment. In case of non contrast-enhancing lesions (i.e., low grade gliomas) FLAIR sequences (TR = 8000, TE = 331.5/7) were also acquired.

**Figure 1:**
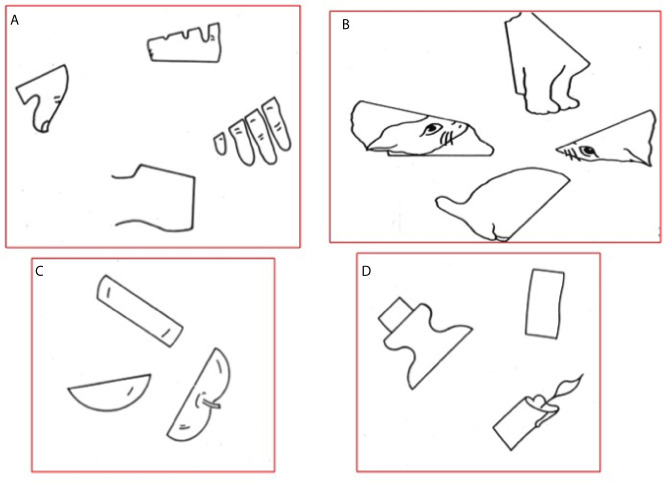
Example of classical drawings showed during the HVOT. **(A)** Hand; **(B)** Cat; **(C)** Apple; **(D)** Candle.

The MRI scan of patients in Group A was imported into the nTMS system for mapping cortical areas involved in the VS network. The nTMS mapping was performed by using the NexSpeech module of the Nexstim NBS 4.3 system (Nexstim Oy, Helsinki, Finland), and basically consisted in the use of repetitive nTMS delivered through a figure-of-eight coil over the scalp of the patient during the execution of the HVOT test. Initially, the resting motor threshold (RMT) for the first dorsal interosseus (FDI) muscle using single-pulse stimulation of the primary motor cortex was defined as previously described (40–42). Then, a repetitive stimulation was applied over both the hemispheres, with particular regard to the parietal lobe and the adjacent frontal, temporal and occipital gyri, during the execution of an nTMS-implemented version of the HVOT test. The HVOT is a standardized test for measuring the individual ability to integrate visual stimuli, and is commonly used during routine neuropsychological assessment for the investigation of visuospatial processing (43). It consists of 30 line drawings depicting simple objects, which have been cut into pieces and rearranged in a puzzle-like fashion (**Figure 1**). The subject is asked to identify what each object would be if all pieces were put back together correctly. All the HVOT drawings were imported into the nTMS system and displayed into a LCD screen in front of the subject. Pictures were presented to subjects for a fixed time (4 s) and with a fixed inter-picture interval (IPI; 4 s). Each participant underwent a baseline task without nTMS stimulation three times, in order to eliminate unrecognized/misnamed drawings, to induce a learning-effect, and therefore to reduce as much as possible false-positive results. Then, the task was administered during the repetitive stimulation. As already reported in literature regarding nTMS mapping of VSAs, the stimulation protocol consisted in a train of 10 pulses with a 5 hz frequency at 100% of the RMT intensity (32, 33). Stimulation intensity was reduced to 90% or 80% of the RMT if the patient complained some discomfort during the mapping procedure. The repetitive stimulation was triggered with the picture presentation by using an onset delay of 0 ms (44). The nTMS coil was randomly moved in about 10-mm steps over the parietal cortex and the adjacent frontal, temporal and occipital gyri. The coil was placed perpendicular to the sulcus posterior to the stimulated point to achieve the maximum field induction (45). During the mapping procedure, about 100 cortical sites in both hemispheres were stimulated 3 times each. Since repetitive nTMS is able to temporarily disrupt brain functions according to a “virtual lesion” model (37, 38), the nTMS mapping induced specific errors during the execution of the HVOT when stimulating cortical areas involved in the VS network. A stimulated cortical site was considered involved in the VS network if an nTMS-induced error at the HVOT was obtained at least during 2 of 3 stimulations. All the procedure was video-recorded and used for the off-line analysis.

### Off-Line Analysis of nTMS Mapping in Group A

The recorded videos of the HVOT performance during both the baseline and the stimulation procedures were accurately analyzed and compared by two experienced neuropsychologists in order to identify nTMS-induced errors. According to the literature, HVOT errors were categorized into performance, part, and language-based errors (46). Performance errors include: 1) *perseverative errors*, consisting in repeating a previous correct or incorrect response on a later item, providing category responses that are unrelated to the current stimulus item but that are related to a previous item; 2) *unformed/unassociated errors*, consisting of a response that is unassociated to the current item, for example, “knife” for “dog”; 3) *don’t know/no response errors*, consisting in providing no response or in do not understand the item. Part errors consist in naming only one part of the current stimulus, for example, “finger” for “hand”. Language-based errors consist of 1) semantically related or unrelated name for an object; 2) circumlocutory response; 3) neologistic response; 4) agrammatic response; 5) incorrect phonemically related response.

Performance and part errors specifically regard visual analytic and synthetic abilities, whereas language-based errors regard the verbal representation of visual stimuli. When an error response occurred during nTMS mapping, the corresponding cortical site was marked as visual-organization related and tagged according to the observed error type (performance, or language-based, or part error).

**Figure 2:**
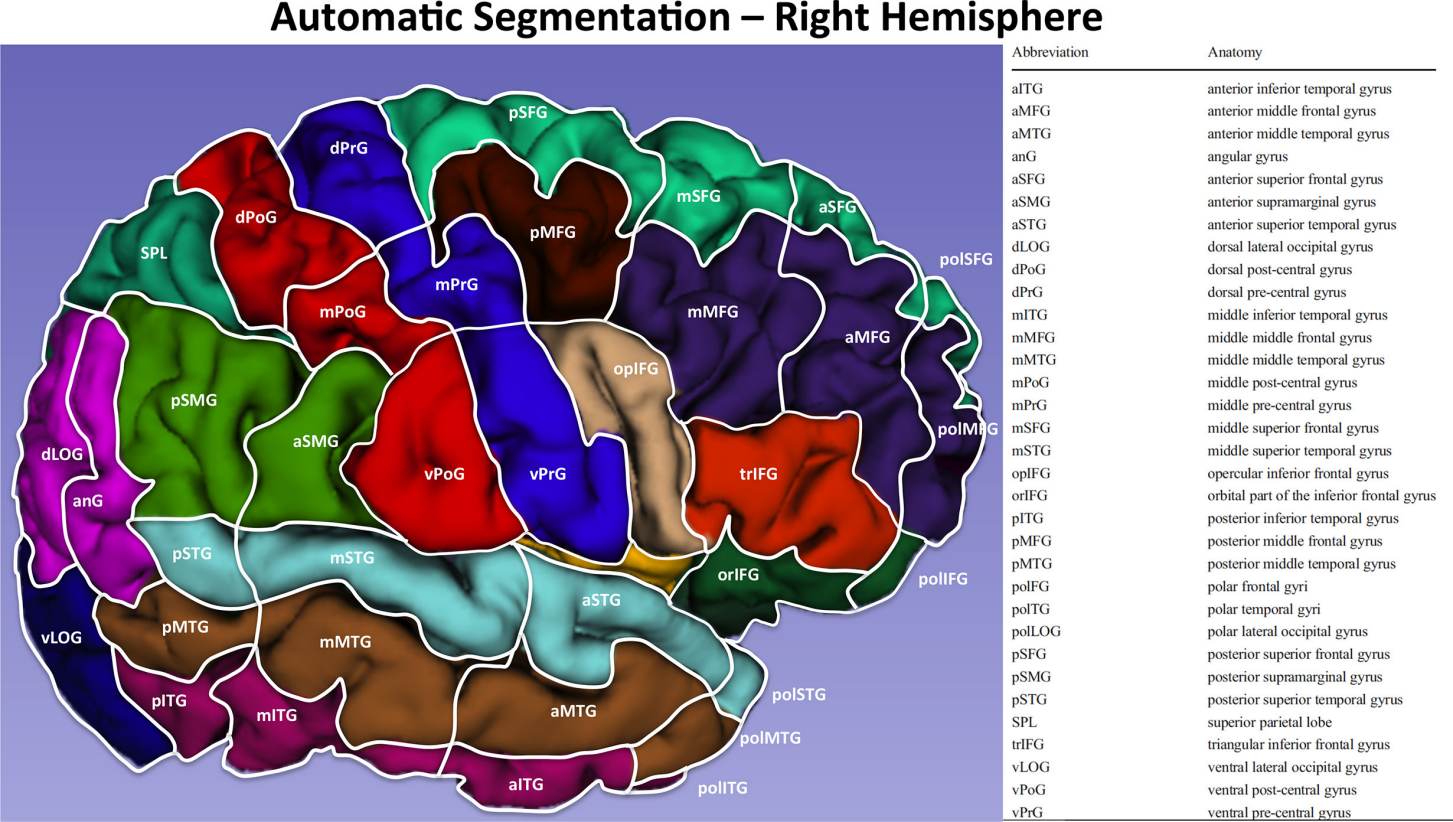
Automatic segmentation of the cortical surface of the right hemisphere according to Corina et al (47). with the relative legend.

After the offline analysis of responses was accomplished, the nTMS cortical spots corresponding to HVOT errors were automatically merged over the patient’s MRI scan and exported as DICOM images (i.e., Fusion MRI scan). Then, the anatomical localization over the brain cortex of each nTMS-induced error was defined. The Fusion MRI scan was used to perform an automatic MRI reconstruction and volumetric segmentation of the brain cortical surface using the Freesurfer image analysis suite, which is documented and freely available for download online (http://surfer.nmr.mgh.harvard.edu/) (**Figure 2**). Then, the Freesurfer surface reconstruction and segmentation of each hemisphere was further segmented according to the cortical parcellation system described by Corina et al. (47, 48). Thereafter, the location of each single nTMS-induced error/spot was identified in a specific Corina’s cortical area.

We analyzed the inter- and intra-hemispheric cortical distribution of the nTMS-induced errors. The errors’ distribution was expressed as error rate % (ER) per single area (number of errors/total stimulation trials) and analyzed in both the right and left hemisphere. As well, the ER distribution was also analyzed according to the different classification of errors (performance, language-based and part errors) in both the hemispheres.

### DTI Tractography of the SLF branches in Group A

During MRI scan acquisition, also diffusion weighted imaging (DWI, 64 directions, TR/repetition time = 2383.9, TE/echo time = 51.9) sequences were acquired for the successive DTI computation. DWI sequences were imported together with the Fusion MRI scan into the Medtronic Planning Station (Medtronic Navigation, Coal Creek Circle Louisville, CO, USA). All the tractography workflow was performed using the StealthViz software (Medtronic Navigation, Coal Creek Circle Louisville, CO, USA). After co-registration of the different sequences, the tensor was calculated, and the software created the Apparent Diffusion Coefficient (ADC) map and the Directionally Encoded Colors (DEC) map. The DEC map was therefore used to choose the ROI for the tractography of the SLF branches. When possible, all the three different components of the SLF (SLF-I, SLF-II, SLF-III) were computed. Otherwise, only the branch closer to the tumor was reconstructed. It is important to highlight that, according to the current literature, the SLF-III is the most ventral portion of the SLF and is synonym of the anterior segment of the arcuate fascicle (49). The tractographic reconstruction was performed choosing a multiple region-of-interest (ROI)-based approach, according to the literature (39, 50), and using a deterministic approach (fiber assignment by continuous tracking algorithm, FACT) with the following parameters: fractional anisotropy (FA) threshold = 0.20; vector step length = 1 mm; minimum fiber length = 50 mm; seed density = 1.0; and max directional change = 55°. In case of massive perilesional edema that could hamper the fiber tracking of the SLF branches, the FA threshold value to stop tracking is progressively reduced in 0.01 steps from the standard 0.20 value up to reach the minimum value of 0.10. If no fibers are visualized, the computation of the specific SLF branch is stopped to avoid false-positive results. Patients were included in the study if at least one of the three SLF branches was successfully computed.

### Preoperative Planning in Group A and Surgical Treatment in Group A and B

In Group A, the nTMS mapping of the VS network and the DTI fiber tracking of the SLF branches were simultaneously visualized into the Neuronavigation System, thus providing a 3D reconstruction of the VS cortico-subcortical network. Such a 3D reconstruction was used by neurosurgeon to plan the best surgical corridor to achieve the maximal tumor resection as well as to preserve as much as possible the VS network. Once the surgical strategy was defined, the 3D reconstruction of the VS network was still displayed during surgery into the neuronavigation system and guided neurosurgeon during tumor resection. The 3D reconstruction of the VS network helped neurosurgeons to spare, as much as possible, the nTMS spots at the cortical level and the SLF branches at the subcortical level.

Surgery was performed under general anesthesia in Group A and B. Nevertheless, in Group B patients, surgical resection was not guided by any preoperative nTMS mapping neither reconstruction of the VS network. In both groups, whenever lesions were located in the anterior parietal lobe and invaded also the frontal lobe, the intraoperative neurophysiological mapping and monitoring of the motor pathway was performed to preserve the motor cortex and corticospinal tract, as previously described (42). Moreover, in case of contrast-enhancing brain tumors, the surgical resection was further guided by intraoperative fluorescence thanks to the administration of intravenous sodium-fluoresceine as we reported elsewhere (51, 52).

### Postoperative Outcome Assessment in Group A and B

In both Group A and B we assessed patients’ outcome by evaluating and comparing the EOR, and the preoperative vs. postoperative 1) functional status expressed through the Karnofsky Performance Status (KPS) score, and 2) visuospatial performances through the HVOT as well as the traditional line bisection task (LBT).

The EOR was assessed in both groups on an early postoperative MRI scan (within 48 hours from surgery). Tumor segmentation and EOR calculation were performed on T1-enhanced sequences or, on FLAIR sequences in case of non-enhancing lesions, using OsiriX Imaging Software^©^ (Pixmeo SARL, Bernex, Switzerland) (53). Segmentation of the tumor was manually performed across all MRI slices (54, 55). The EOR was defined as the difference between the preoperative and postoperative tumor volumes (ml) (56). The EOR was defined as follows: gross total resection (GTR) = no residual pathological tissue; subtotal resection (STR) = less than 10 ml of pathological tissue residue; partial resection (PR) ≥ 10 ml of tissue residue; biopsy ≥ of 95% of tumor residue (42, 57, 58).The EOR was expressed describing the percentage of GTR in both groups.

The KPS was evaluated before surgery and after one month from surgical treatment.

The preoperative neuropsychological evaluation was performed before surgery by two experienced neuropsychologists and included a general assessment of the VSAs by administering the standard LBT and HVOT. Moreover, during the preoperative evaluation, the hemispheric language dominance was assessed according to the handedness defined through the Edinburgh Handedness Inventory (EHI) (59). Post-operative visuospatial outcome was assessed at discharge and at one month from surgery during a standard neuropsychological evaluation including the assessment of VSAs through the administration of the standard LBT and HVOT. The HVOT and LBT scores at the one month follow-up were compared with the corresponding preoperative scores. The HVOT performance was expressed as a T-score (range 41-104) (43, 46, 60). The higher is the T-score, the higher is the probability of VSAs impairment. The LBT score was expressed according to the current literature (61).

### Statistical Analysis

The paired Student T-test was used for the analysis and comparison of the inter- and intra-hemispheric distribution of the ER, as well as for the comparison of the pre- vs. postoperative KPS, HVOT, and LBT scores in each group. The unpaired Student T-test was used to compare different quantitative parameters, including clinical characteristics and outcome findings in Group A vs. Group B. The one-way ANOVA with Tukey post-hoc correction for multiple comparisons was used to compare the mean ER in each hemisphere according to the different error type (performance, language-based, part). Finally, contingency table with Chi-square or Fisher test were used to compare qualitative parameters in Group A vs. Group B, as well as to investigate the association between the eloquence defined by the nTMS-based planning and the EOR in group A. Statistical significance was defined as a *p* value < 0.05. Data analysis was realized by using GraphPad Prism version 6.00 for Windows, GraphPad Software, La Jolla, California, USA, www.graphpad.com.

## Results

### Demographic and Clinical Characteristics of Patients in Group A and B

The Group A included a total of 20 patients (10 males, 10 females, mean age 58.35 ± 14.03). All patients were monolingual (native-language: Italian) and right-handed. Tumors were mainly located in the right parietal lobe in all cases. Nevertheless, in 8 cases the neoplastic tissue invaded also the ipsilateral frontal lobe, in 4 the temporal lobe, and in 2 cases the occipital lobe. Pathological examinations revealed that 16 patients were affected by glioblastoma (GBM), 3 by diffuse astrocytomas, and 1 by lung cancer metastases. The mean preoperative KPS was 83.5 ± 6.7. The preoperative neuropsychological evaluation showed that the mean T-score computed through the HVOT was 70.85 ± 8.08, while the mean LBT score was 7.15 ± 0.87.

The Group B included 20 patients (12 males, 8 females, mean age 58.65 ± 11.47). As well as in Group A, all patients were monolingual, Italian native speakers, and right-handed. Tumors were all manly located in the right parietal lobe, and involved also the frontal lobe in 7 cases, the temporal lobe in 5, and the occipital lobe in 2. The histological diagnosis was GBM in 13 cases, diffuse astrocytoma in 4, anaplastic astrocytoma in 1, metastases from lung cancer in 1, and from breast cancer in the remaining one. The mean preoperative KPS was 84 ± 6.8. The mean T-score at the HVOT was 71.6 ± 5.49, and the mean LBT score was 7.1 ± 0.78.

**Table 1** shows salient demographic and clinical characteristics of patients in Group A and B, as well as nTMS mapping features of patients in Group A. Statistical analysis showed no significant differences in Group A vs. Group B for all the preoperative demographic and clinical characteristics.

### nTMS Cortical Mapping of VSAs in Group A

In Group A, the nTMS mapping of cortical areas involved in the VS network was feasible and well tolerated in all cases. The mean RMT was 36.85 ± 6.2 μV in the right hemisphere and 35.3 ± 3.97 μV in the left hemisphere (**Table 1**). The difference was not statistically significant.

**Figure 3:**
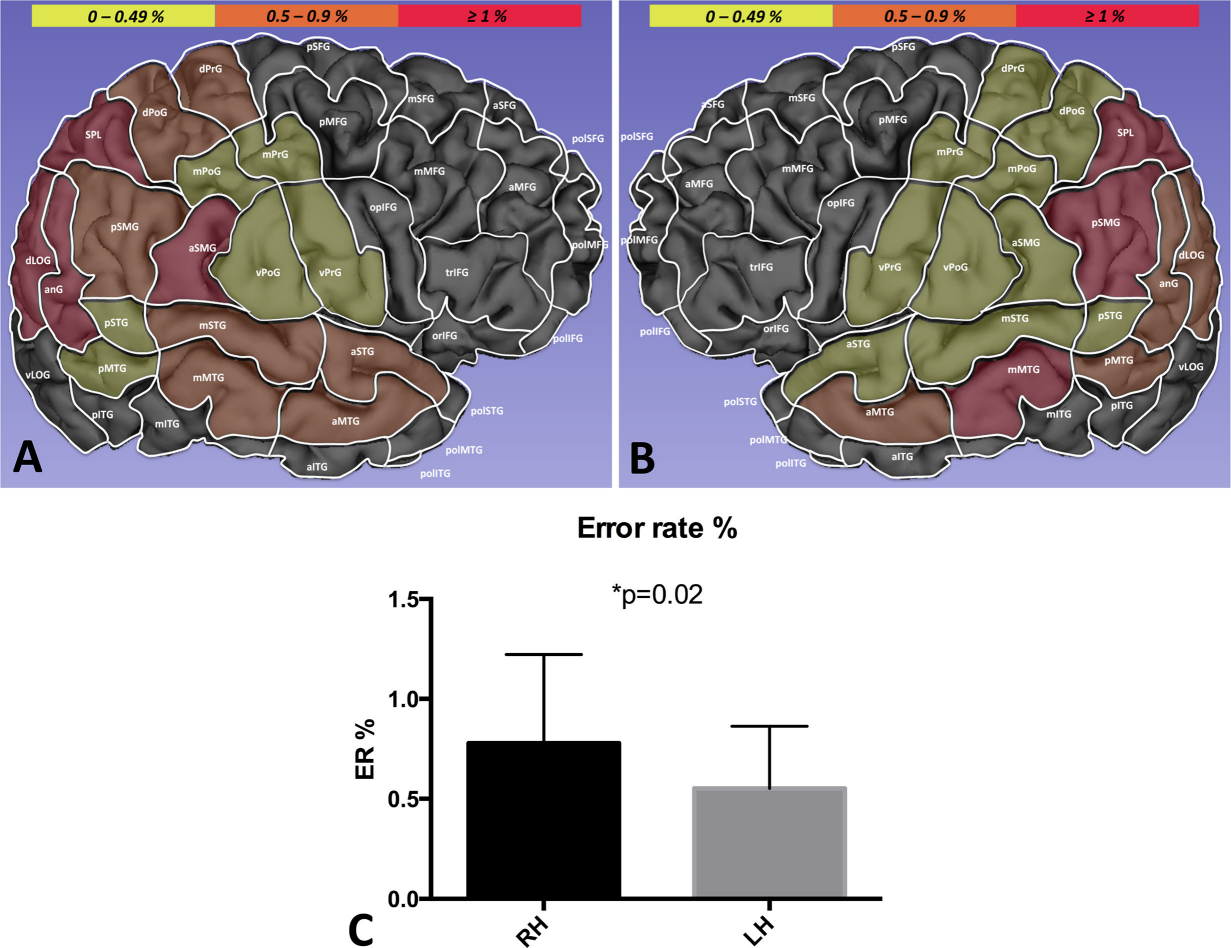
Distribution of the global ER in the right **(A)** vs. left hemisphere **(B)**. The ER (%) in each single area is identified by a growing color intensity (yellow, orange, red). The ER was significantly higher in the right hemisphere (RH) as compared to the left hemisphere (LH), suggesting a lateralization of VSAs **(C)**.

The offline analysis of the nTMS-induced errors showed that the ER was significantly higher in the right hemisphere vs. the left one (0.77% ± 0.44 vs. 0.55%± 0.31, p=0.02) (**Figure 3C**)

In the right hemisphere, the ER was higher in the anterior supramarginal gyrus (aSMG, 1.7%), angular gyrus (anG, 1.4%), superior parietal lobule (SPL) (1.3%), and dorsal lateral occipital gyrus (dLoG) (1.2%)(**Figure 3A**). The analysis of the intra-hemispheric ER distribution according to the different type of errors showed that performance errors were significantly more frequent then language-based and part errors (respectively, 0.34% ± 0.31 vs. 0.15% ± 0.15 vs. 0.28% ± 0.12, p=0.02).

Conversely, in the left hemisphere, the ER was higher in the SPL (1.14%), posterior supramarginal gyrus (pSMG, 1.12%), and middle superior temporal gyrus (mSTG, 1.04%). (**Figure 3B**). The intra-hemispheric analysis of the cortical distribution of errors showed the language-based errors were induced more frequently than performance and part errors (respectively, 0.29% ± 0.17 vs. 0.16% ± 0.17 vs. 0.09% ± 0.09, p=0.001).

**Figure 4:**
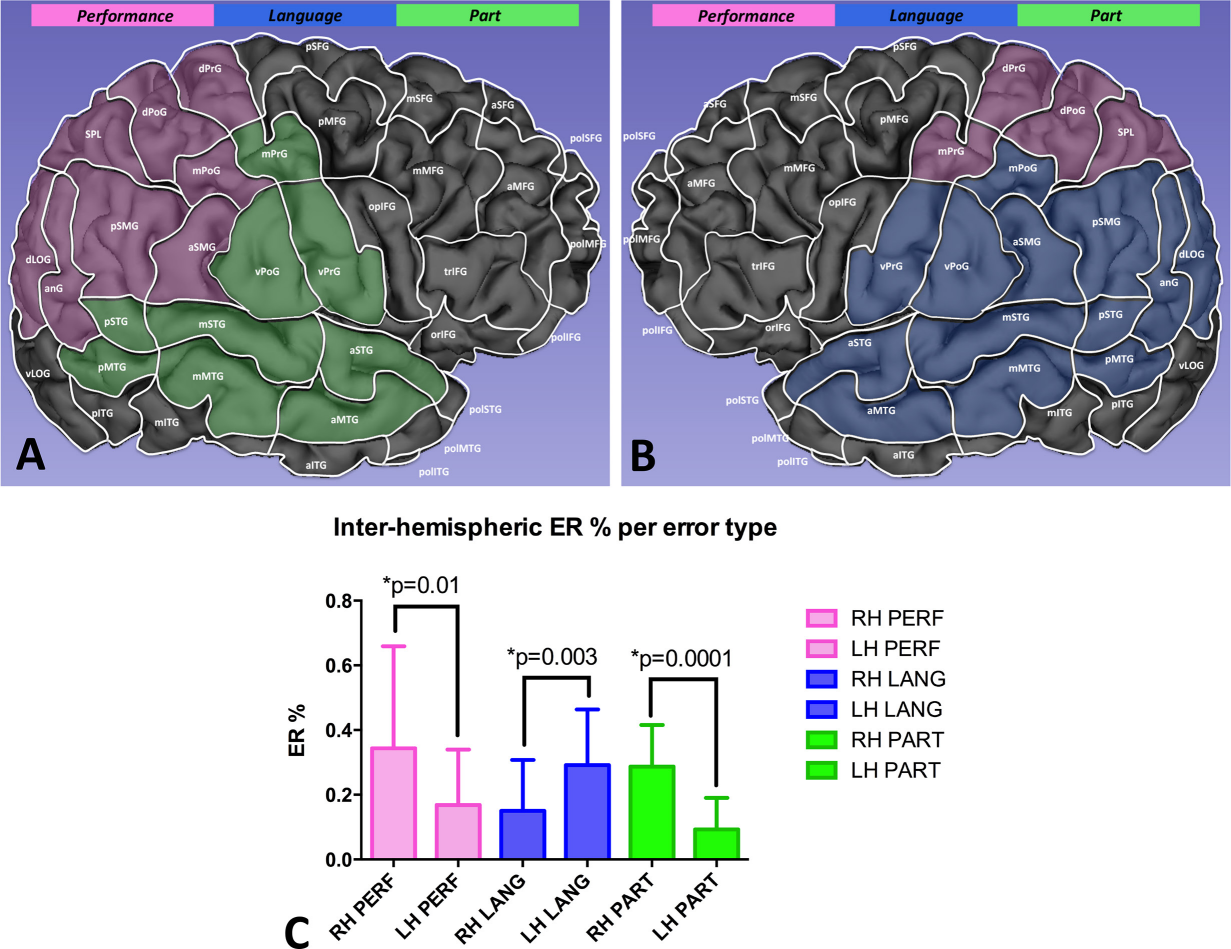
Comparison of the ER per error type between the right **(A)** and the left hemisphere **(B)**. Performance and part errors were significantly more frequent in the right hemisphere, while language-based errors occurred more frequently in the left hemisphere **(C)**.

The analysis of the inter-hemispheric cortical distribution of the ER showed that performance and part errors were significantly higher in the right hemisphere as compared to the left one (respectively, 0.34% ± 0.31 vs. 0.16% ± 0.17, p=0.01; 0.28% ± 0.12 vs. 0.09% ± 0.09, p=0.0001). Conversely, language-based errors were significantly more frequent in the left hemisphere (0.29% ± 0.17 vs. 0.15% ± 0.15, p=0.003) (**Figure 4**).

### Preoperative Planning and Surgical Resection in Group A

**Figure 5:**
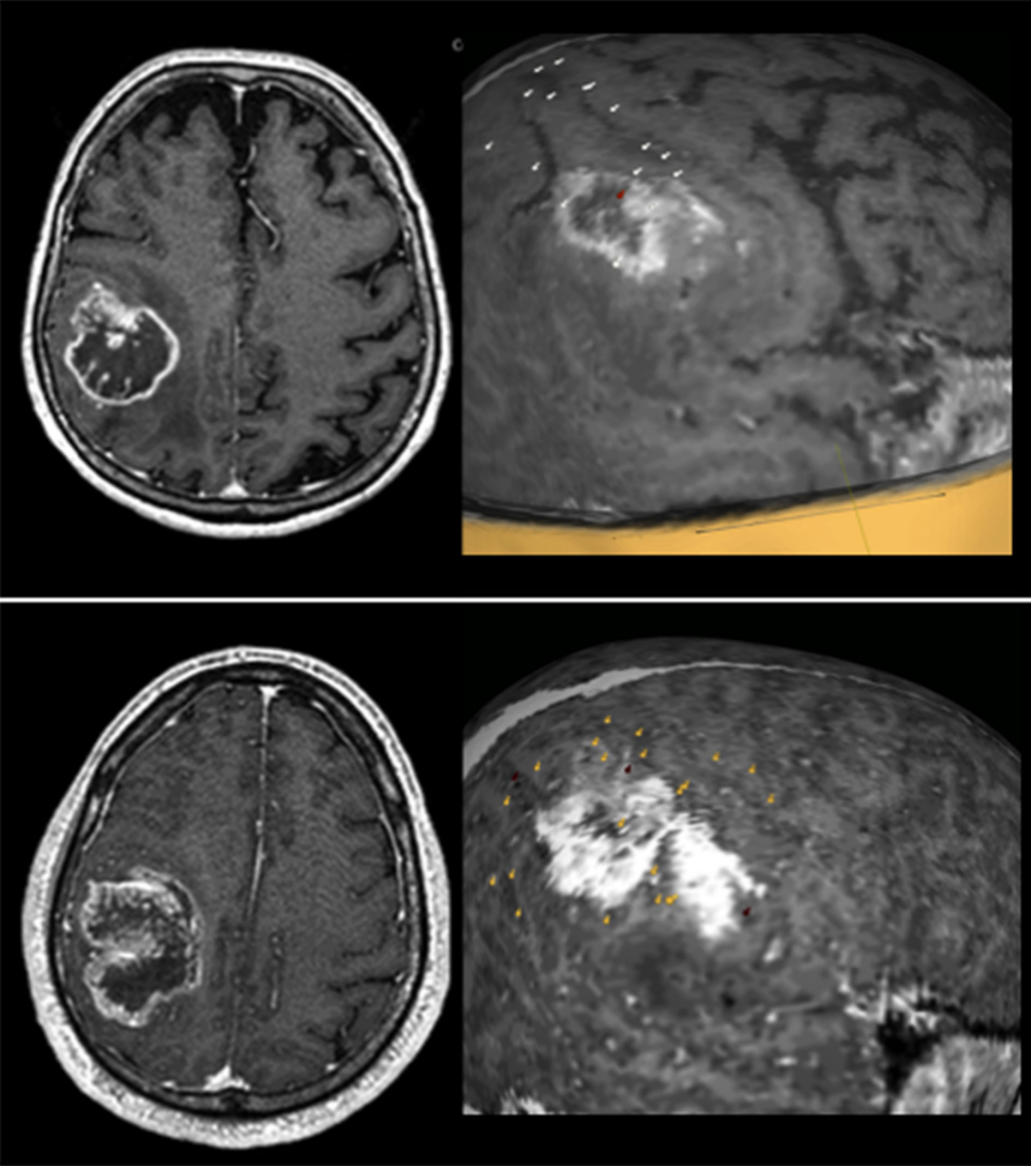
Some examples of the nTMS mapping of VSAs in patients with tumors involving the right parietal lobe. In all three cases some nTMS spots are overlapped to the lesions, suggesting a high risk for postoperative VSAs deficits. Spots are color-coded: white = performance errors; red = language-based errors; yellow = part errors.

**Figure 6:**
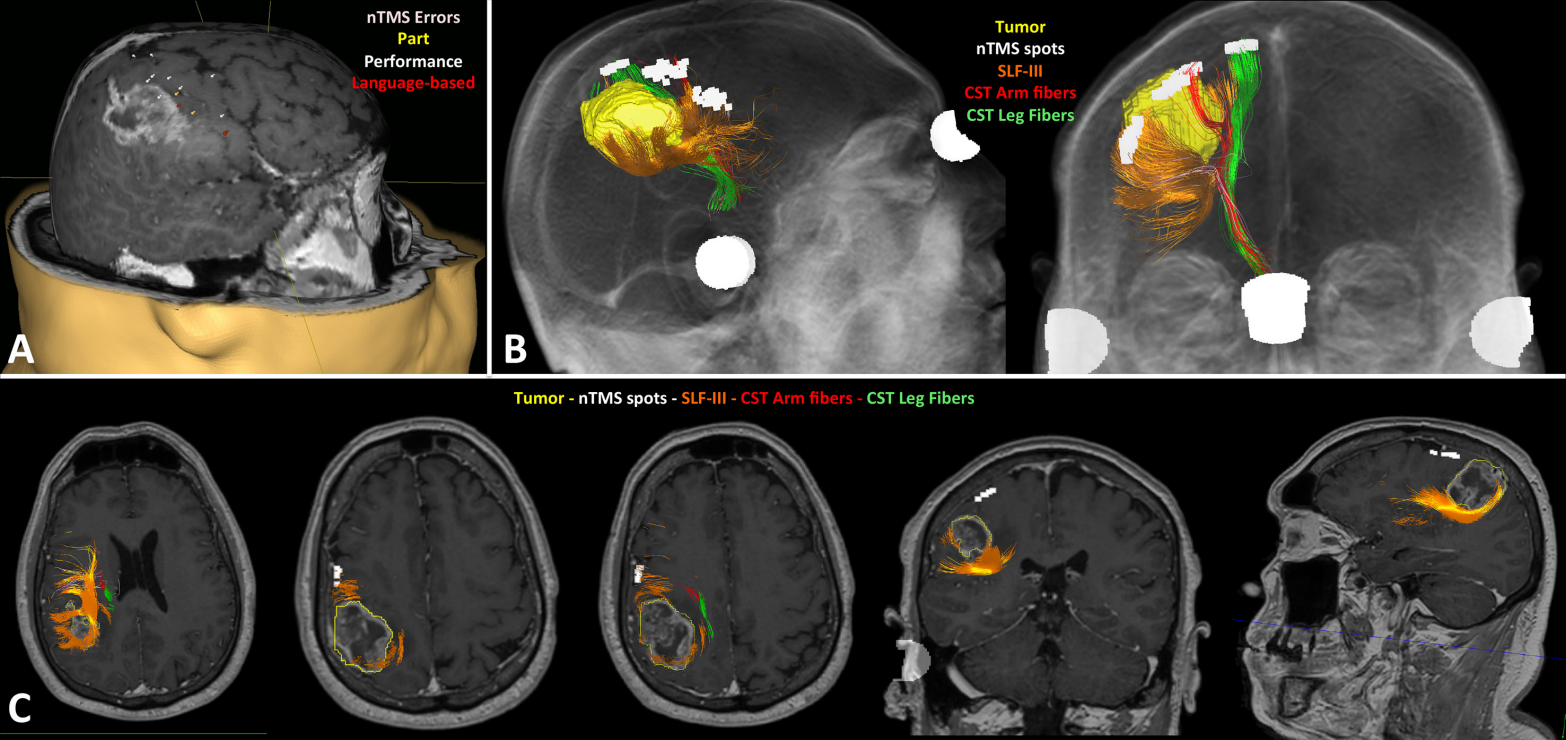
Case example of the preoperative planning in a right fronto-parietal glioblastoma. The nTMS mapping of VSAs identified several eloquent cortical sites of the VS network **(A)**; the DTI tractography showed the tumor (yellow) was very close to the SLF-III (orange), but also to the corticospinal tract (CST; arm fibers in red, leg fibers in green) **(B)**; the fusion MRI scan confirmed the SLF-III was infiltrated by the posterior part of the tumors, as visible in the different axial, coronal and sagittal slices; conversely, the nTMS cortical spots are far away from the tumor **(C)**.

**Figure 7:**
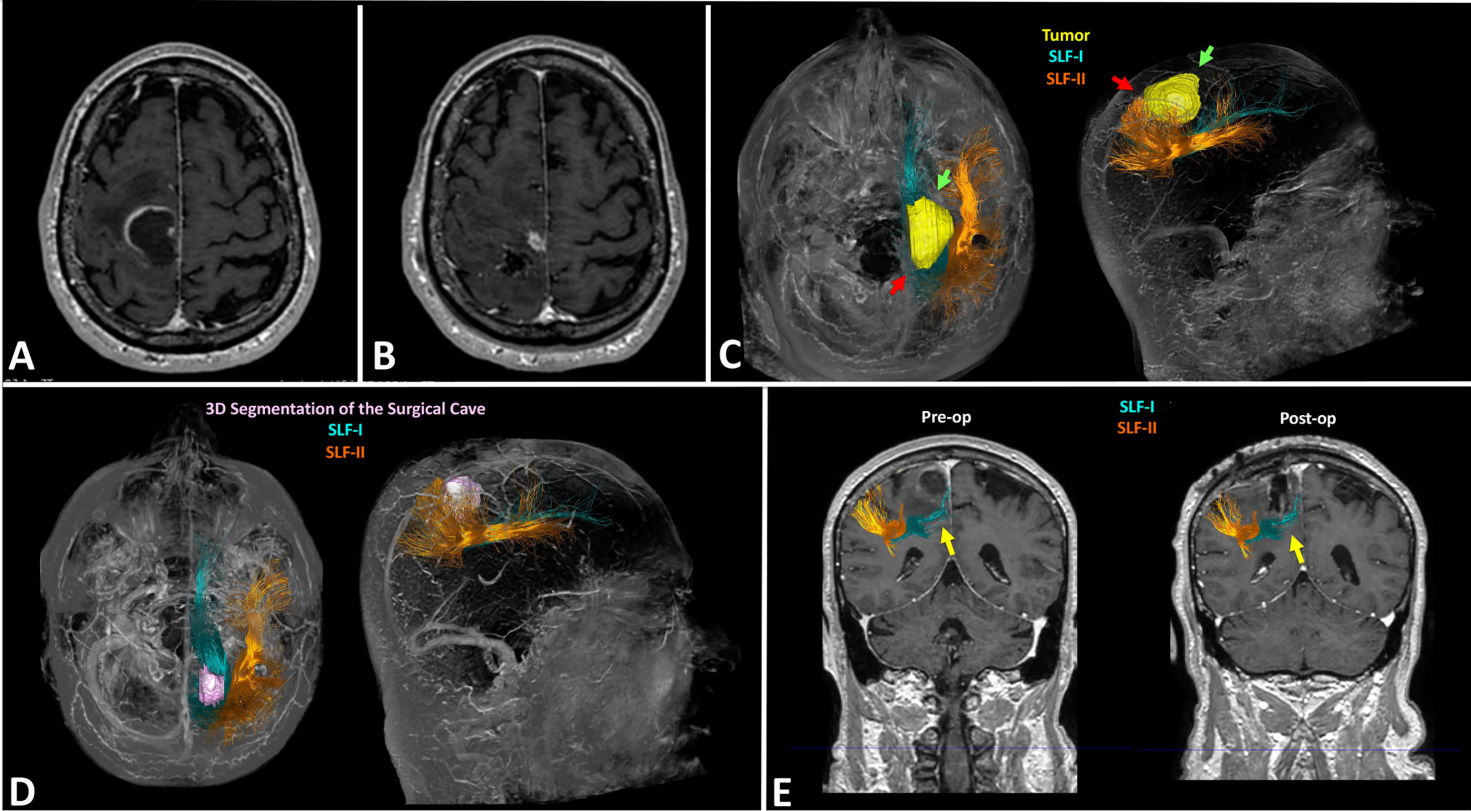
Case example of the planning in a case of right fronto-parietal GBM (case 4 in **Table 1**). Preoperative MRI scan documenting the lesion is located in the anterior portion of the right parietal lobe and infiltrates also the primary motor cortex **(A)**; postoperative MRI scan documenting the subtotal resection of the lesion: neurosurgeons removed only the portion located in the right parietal lobe, while they didn’t resect the portion infiltrating the motor cortex **(B)**; preoperative reconstruction of the VS network showing the posterior portion of the lesion is close to the blue fibers of the SLF-I indicated by the red arrows: this induced a change of surgical strategy leading neurosurgeons to start resection from the antero-lateral portion of the tumor indicated by the green arrows, just medially to the orange fibers of the SLF-II **(C)**; postoperative DTI fiber tracking showing the 3D rendering of the surgical cave in pink, and the preserved blue fibers of the SLF-I and orange fibers of the SLF-II **(D)**; coronal section showing the preservation of the SLF-I and II, especially the blue fibers of the SLF-I running medial to the surgical cave **(E)**.

**Figure 8:**
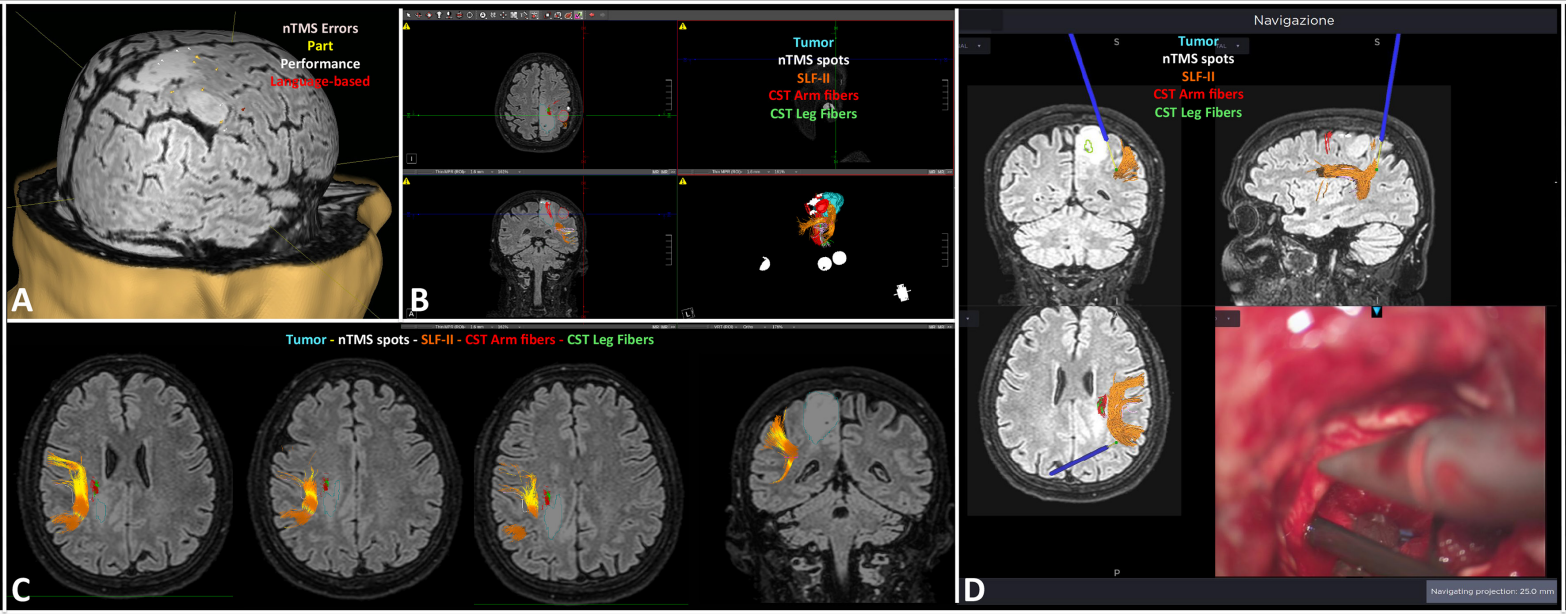
Case-example of the intraoperative use of the 3D reconstruction of the VS network in a case of right parietal diffuse astrocytoma. The nTMS mapping documented some spots surrounding the lesion, and two overlapped to it **(A)**; a snapshot of the preoperative planning describing the relationship between the tumor and the VS network **(B)**; the axial and coronal slices show the proximity of the tumor (light blue) to the SLF-II (orange) and the corticospinal tract (CST; arm fibers in red, leg fibers in green) **(C)**; example of the intraoperative verification of the distance between the tumor, the navigation pointer (blue stylet) and the SLF-II (orange) **(D)**.

In Group A patients, the nTMS cortical mapping (**Figure 5**) and the DTI tractography of the SLF (one or more of the three major branches) were successfully combined in all cases, thus providing a 3D representation of the VS network. The preoperative reconstruction of the VS network and the analysis of its spatial relationship with brain tumors enabled to identify a concrete risk for injury to the network during surgery in 8 out of 20 cases (40%). Such a risk was considered concrete because of the proximity of the tumor (≤10mm) (42, 58) to the nTMS cortical spots and/or the SLF branches. Eight lesions close (≤10mm) to the VS network were considered true-eloquent; the remaining 12 were defined as false-eloquent (12 out of 20). Such a preoperative risk stratification of patients was used to plan the best-customized surgical approach to preserve the components of the VS network (**Figure 6**). In some cases, the visualization of the preoperative reconstruction of the VS network induced a change of the original surgical strategy hypothesized before by neurosurgeons (**Figure 7**). After a definitive surgical plan had been established, surgery was performed under the guidance of the 3D reconstruction of the VS network in all cases. Indeed, it was continuously visualized into the neuronavigation system and guided the neurosurgeon in performing tumor resection and preserving the nTMS spots and the SLF (**Figure 8**).

### Comparison of Outcome Variables in Group A vs. Group B

**Figure 9:**
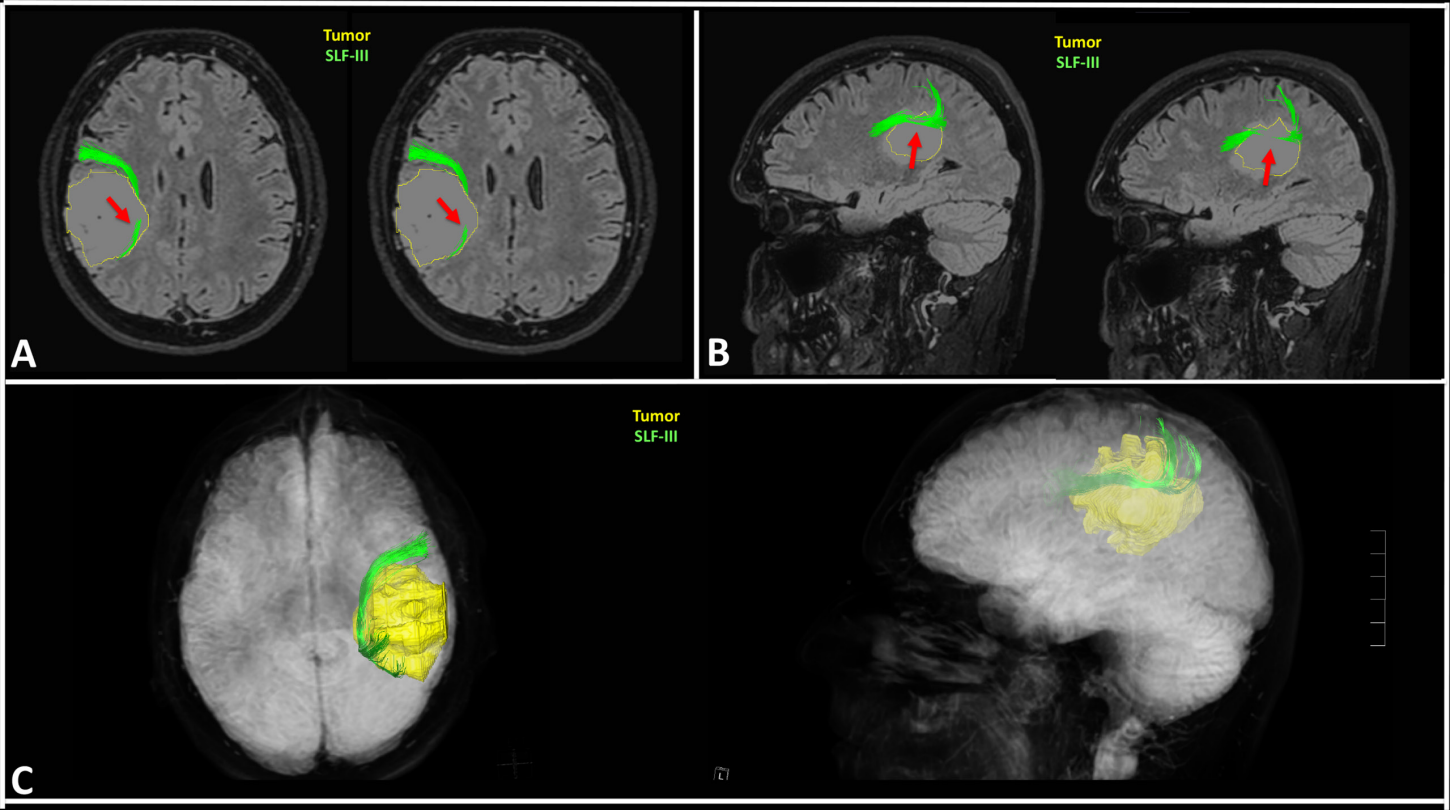
Case example of the planning in a case of right fronto-parietal diffuse astrocytoma. The preoperative 3D reconstruction of the SLF-III showed it was infiltrated by the tumor. The green fibers of the SLF-III are infiltrated by the medial **(A)** and superior **(B)** portion of the tumor as indicated by the red arrows; the 3D reconstruction confirmed the infiltration of the SLF-III, thus inducing neurosurgeon to plan a subtotal resection to preserve the VS network **(C)**.

In Group A the GTR was achieved in 17 out of 20 patients (85%). In all cases the neurosurgeon performed the resection under the neuronavigation guidance up to the complete removal of the neoplastic tissue close to the structures of the VS network. Only in three cases 3 cases (15%) a STR was obtained because of the proximity of the tumor to the corticospinal tract and the primary motor cortex. In one of these 3 cases, the tumor infiltrated also the SLF and the neurosurgeon decided to plan a subtotal resection and to leave a small portion of the SLF-infiltrating neoplastic tissue (**Figure 9**). In the remaining 19 cases the proximity of the tumor to the nTMS spots and/or the SLF branches never required the interruption of resection. Statistical analysis through the Fisher test documented a slightly significant association between the eloquence defined by the nTMS-based planning (true vs. false) and the EOR (GTR vs. STR) (p=0.04).

**Figure 10:**
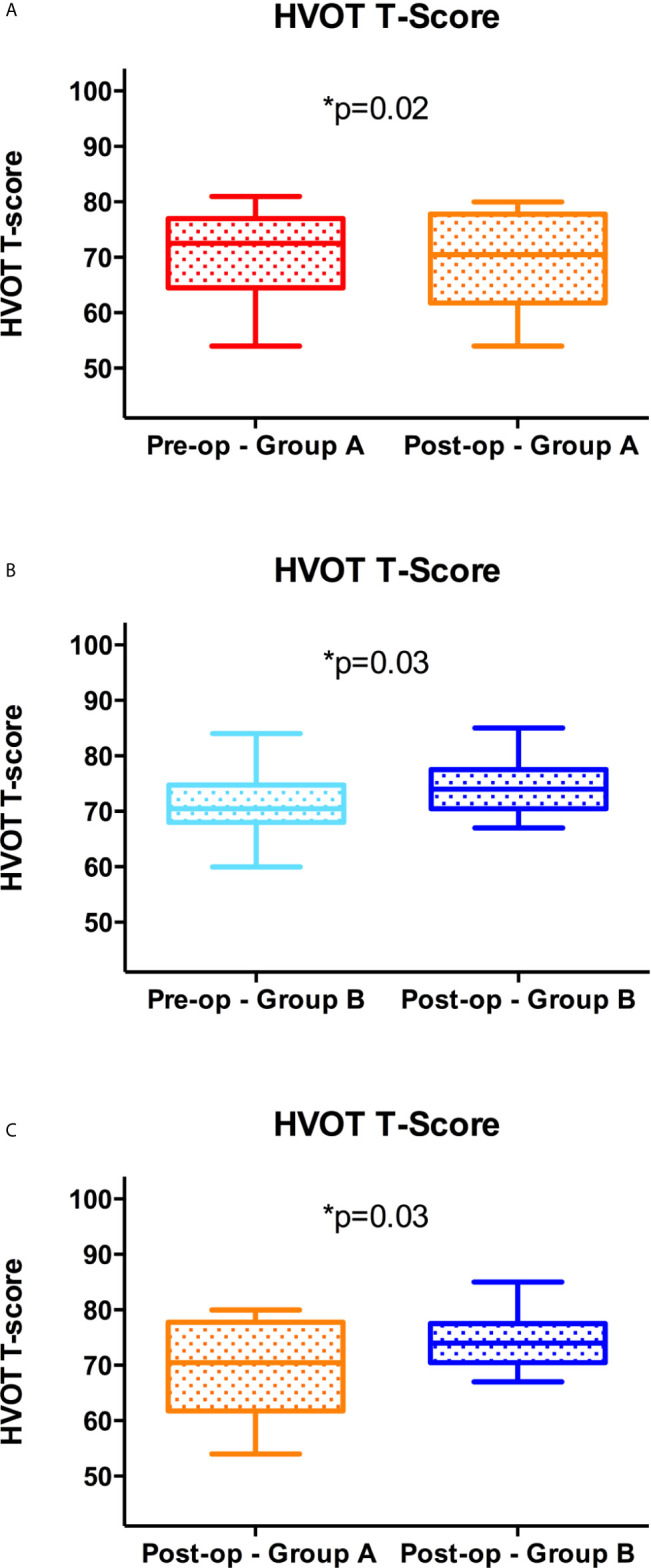
Visuospatial outcome at one month from surgery. Significant reduction of the postoperative vs. preoperative T Score at the HVOT in Group A **(A)**; significant increase of the postoperative vs. preoperative T Score at the HVOT in Group B **(B)**; significant reduction (improved VSA) of the postoperative T Score at the HVOT in Group A vs. Group B **(C)**.

At discharge, no new deficits of VSAs were observed in the study population during the standard neuropsychological evaluation. At one month from surgery, we observed a significant reduction of the T-score at the HVOT (69.60 ± 8.21 vs. 70.85 ± 8.08; p=0.02), and an improvement of the LBT score (7.15 ± 0.87 vs. 7.4 ± 0.94; p=0.05) suggesting an improvement of VSAs as compared to the preoperative period (**Figure 10A**). After one month from surgery, we recorded a stable KPS score as compared to the preoperative period (83.5 ± 8.1 vs. 83.5 ± 6.7; ns).

In Group B, the GTR was achieved in 18 out of 20 patients (90%). No preoperative stratification of the risk for the VS network was available before surgery. After one month from surgery, the T-score at the HVOT was significantly increased (74.3 ± 4.53 vs. 71.6 ± 5.49; p = 0.03), while the LBT score was significantly reduced (6.6 ± 0.75 vs. 7.1 ± 0.78; p=0.01), suggesting a worsening of VSAs as compared to the preoperative period (**Figure 10B**). Finally, at one month from surgery, we observed also a non-significant worsening of the KPS score (82.5 ± 9.1 vs. 84 ± 6.8; ns).

The comparison of outcome parameters between the two groups, documented a significant improvement of the postoperative T-Score at the HVOT (p = 0.03) and of the LBT score (p = 0.005) in Group A as compared to Group B (**Figure 10C**). No significant differences were found for the EOR and KPS score comparing the two groups.

## Discussion

Surgical treatment of brain intrinsic tumors aims to the maximal resection of the neoplastic tissue and to the simultaneous preservation of the adjacent functional brain networks to reduce postoperative morbidity that could seriously affect the functional independence and quality of life of patients (62). Such an objective has been translated in an increasing ability of neurosurgeons to preserve especially the motor and language network, thanks also to the availability of innovative technologies and surgical strategies. Nevertheless, in the recent years a growing attention has been paid also to the preservation of other complex brain functions, including VSAs (8). In fact, a surgical damage to the brain networks involved in these functions during brain tumor resection could seriously affect the patients’ quality of life. VSAs rely on a complex fronto-parietal network that shows a significant lateralization to the right hemisphere (19, 46). Accordingly, brain tumors located within the right parietal lobe can cause a VASs impairment (24, 63). Nevertheless, VSAs impairment is usually underestimated or not properly evaluated in neurosurgical patients because of the need of a specific neuropsychological expertise (8). Many neurosurgical departments have recently developed new strategies for the assessment of VSAs in brain tumor patients, trying to reduce the occurrence of new postoperative deficits. Among these, the intraoperative neurophysiological mapping (IONM) of the VS network during awake surgery seems to be the most effective (10). As a matter of fact, IONM is considered the gold standard technique for resection of CNS tumors (64–67). Nevertheless, not all patients are eligible for awake surgery (22, 23). A good alternative is represented by the preoperative mapping of the VS network using advanced neuroimaging techniques. Among these, nTMS has recently gained great favor in the neurosurgical community (68). It allows for a non-invasive identification of eloquent cortex prior to surgery, including motor and language areas. Several studies reported that nTMS mapping improves surgical treatment and outcome of patients affected by brain tumors in eloquent areas (34, 58, 69, 70). Moreover, nTMS can be successfully combined with DTI tractography in the clinical practice, thus enabling the visualization of eloquent networks and the analysis of their spatial relationship with the tumor: that allows for a customized preoperative planning that guides neurosurgeons to achieve the maximal safe resection of brain tumors in critical areas (34, 71, 72). Nevertheless, to our knowledge no studies have ever reported the use of nTMS mapping in combination with DTI tractography for the reconstruction of the VS network, with the final aim to improve surgical treatment of brain tumors involving such a network and reduce the occurrence of postoperative impairment of VSAs.

In the present study, we reported our preliminary experience using the nTMS cortical mapping of the VS network, in combination with the DTI tractography of the SLF branches. Previous studies reported the possibility to use repetitive nTMS for mapping VSAs. Giglhuber K. et al. in a first paper in 2016 reported a novel approach to evoke neglect-like symptoms in healthy subjects using repetitive nTMS. They implemented a line bisection judgement task (the landmark task) in the nTMS system to map cortical areas involved in the VS network. They found a higher nTMS-induced error rate in the aSMG, dLOG, and SPL (32). In a second paper from the same authors, in 2018, repetitive nTMS was used in healthy subjects to map visuospatial attention (33). In this study, using the greyscale task (73), the authors found that nTMS was able to induce leftward or rightward deviations of the VS attention by stimulating the right hemisphere, especially the pSMG, SPL, anG, vLOG, several temporal (mSTG, pMTG mMTG) and frontal areas (mSFG, mMFG, pMFG, mMFG, trIFG, opIFG). The most interesting finding reported is that repetitive nTMS is a feasible technique to map the cortical component of the VS network. Nevertheless, these experimental studies were performed only in healthy subjects. The most important result of our study is the confirmation of preliminary results reported by Giglhuber K. et al. in patients affected by tumors located in the right parietal lobe. As a matter of fact, we found a higher ER in the same areas of the right hemisphere reported in previous studies: the aSMG, anG, SPL, and dLOG. However, many error responses were also evoked by stimulating the temporal lobe, in particular the superior temporal gyrus (aSTG, mSTG, pSTG), like it has been reported in previous nTMS studies (32). Since we performed mapping in brain tumor patients, we explored only the parietal lobe and the adjacent temporal, frontal and occipital gyri: we didn’t applied nTMS over the most anterior portion of the frontal lobe and therefore we cannot confirm or deny the ability of repetitive nTMS to map the frontal cortical areas involved in the VS network as reported in the second study by Giglhuber et al. (33).

The results of our nTMS-based mapping of cortical regions belonging to the VS network are also concordant with previous studies based on lesional models or intraoperative brain mapping that indicated the right inferior and superior parietal, angular, and middle occipital cortices are key anatomical structures in the visuoconstruction processes (17–20). Furthermore, evidence from previous standard TMS studies in healthy subjects showed that the right posterior parietal cortex (rPPC), right supramarginal gyrus, and medial SPL are involved in visuospatial localization (74), visual search tasks (75, 76), and general visual selection mechanisms (77).

Interestingly, we achieved similar results using the same repetitive nTMS protocol of Giglhuber K. et al. but a different neuropsychological task, that is the HVOT. HVOT is commonly used during the routine neuropsychological examination to measure visuospatial processing (78–83). The cognitive processes investigated by HVOT are multifactorial, including mental rotation, visual working memory, object identification and name retrieval (78). Although some authors suggested the HVOT is not a pure visuospatial test, but implicates other functions such as language (84), it has been reported that it clearly loads on a global dimension called *non-verbal cognitive ability* which encompasses a large variety of attentional and visuospatial measures (85). Nadler J. et al. reported a simply method for qualitative analysis of HVOT results that is based on the distinction between errors related to visuospatial processing (performance and part errors) and errors related to language functions (language-based errors) (46). In their paper the authors clearly documented a lateralization of different error types, being part and performance errors more common in patients with lesions in the right hemisphere, while language-based errors occurring more frequently in patients with left hemisphere lesions. Interestingly, using the nTMS-implemented version of the HVOT we observed a significant different inter-hemispheric distribution of the nTMS-induced errors: performance and part errors occurred more frequently in the right hemisphere (p=0.0001), while language-based were more commonly induced by stimulating the left one (p=0.003). Such findings exactly confirm results of Nadler et al. in their lesional model, and are concordant with the current knowledge about the location of the visuospatial and language networks (19, 25, 46). Therefore, the qualitative analysis of the HVOT error responses (even those nTMS-induced) allow to discriminate language-based errors related to the impairment (permanent, like in cases of stroke, or transient, like in case of nTMS stimulation) of the language network from part and performance errors due to the impairment of the VS network. Accordingly, in our study, cortical areas showing a higher nTMS-induced ER of language-based errors were exclusively located in the left hemisphere (**Figure 4**).

The neuroanatomic correlates of the HVOT have been investigated by Moritz C.H. et al. (78) using an fMRI-implemented version of the test in a cohort of healthy subjects. The authors found fMRI activation of the bilateral SPL,bilateral lateral occipital and posterior medial temporal lobes, bilateral middle frontal gyri and left anterior cingulate gyrus, with a strong right lateralization. These findings are concordant with the results of our study and with those of Giglhuber K. et al (32, 33), thus demonstrating that nTMS is able to accurately map cortical areas involved in the VS network in the posterior parietal, occipital and frontal cortex.

Nevertheless, the identification of cortical areas involved in the VS network is not enough to preserve the network during brain tumor surgery, thus avoiding the occurrence of postoperative VSAs impairment. Tractography studies largely demonstrated the role of the SLF in the VS network (11–14, 39, 86). The different branches namely SLF-I, SLF-II and SLF-III connect the posterior parietal cortex to the dorsal and ventral frontal cortex, thus creating a complex fronto-parietal VS network (13, 39). Such a subcortical network must be preserved during brain tumor surgery to avoid the occurrence of new postoperative VSAs deficits. In the literature, there are few reports on the use of IONM during awake surgery to preserve VSAs during brain tumor surgery (10, 20, 87). Nevertheless, to our knowledge, there are no reports about the use of preoperative functional imaging techniques that could be useful to preserve VSAs in patients operated for brain tumors located within the right parietal lobe. In the present study, we report for the first time the combination of nTMS cortical mapping and DTI tractography for the preoperative 3D reconstruction of the cortico-subcortical structures of the VS network in patients affected by right parietal lobe brain tumors. Such a reconstruction was successfully used to plan and guide a safe surgical strategy to achieve the maximal tumor resection while respecting the cortico-subcortical component of the VS network (**Figure 7**). In the present study, the first result of the availability of the 3D reconstruction of the VS network for preoperative planning was that neurosurgeons were able to identify true-eloquent lesions (40% of cases) characterized by their proximity/infiltration of nTMS spots (indicating cortical eloquent sites for VSAs) and/or the SLF branches: in this cases a more careful tumor resection was planned to preserve the VS network. On the other hand, false-eloquent tumors (60%) located far from the VS network were approached more aggressively. Resection was stopped in 3 cases because the infiltration of the motor pathway, and in 1 of these cases the infiltration of the SLF induced a further, small limitation of the extent of tumor resection. Nevertheless, in this case the EOR would have been anyway subtotal because of the infiltration of the motor pathway.

The final result of such a surgical strategy based on a specific attention paid to the VS network during tumor resection was that no cases of worsened performance at the HVOT or at a standard VS test such as the LBT were observed after one month from surgery. Moreover, the comparison with a matched historical control group documented that the availability of the preoperative reconstruction of the VS network for planning and guiding surgical resection led to a significant improved postoperative VS performance at the HVOT and LBT in comparison with the standard microneurosurgical resection during asleep surgery. Nevertheless, no significant differences were found comparing the EOR, thus suggesting that our proposed strategy could be helpful specifically to preserve the VS network during surgery without reducing the possibility to achieve the GTR of the tumor. Such a strategy could also potentially have a positive impact on the postoperative KPS score, although we were not able to document any significant difference between the two study groups. However, patients treated without using the nTMS-based mapping and planning showed a non-significant reduction of the postoperative KPS score as compared to the preoperative period.

Similar surgical strategies have been already reported for resection of tumor close to the motor or language networks: several studies (including some from our group) reported that the use of a preoperative planning based on nTMS cortical mapping and nTMS-based DTI tractography is associated to a tailored less-invasive surgical approach, and to an improved EOR and functional outcome (23, 42, 58, 69, 88, 89). Since this is the first study evaluating the impact of such an advanced preoperative planning for the 3D reconstruction of the VS network in brain tumor patients, it is plausible to speculate that this approach could lead to those encouraging results in terms of improvement of surgical treatment and outcome already observed for the nTMS-based planning in patients with motor- or language-eloquent brain tumors. Nevertheless, although the results of our study are encouraging, further larger prospective studies are needed to confirm or deny our preliminary observations.

### Limitations of the Study

The single-center retrospective design, the small number of patients enrolled, and the comparison with a matched historical control group limit the strength of our conclusions. Moreover, this study suffers from the common intrinsic limitations of nTMS, such as the different mapping accuracy due to the use of different stimulation parameters (44, 90, 91), but also the inaccuracy of the nTMS navigation during both the registration and the stimulation phases (92, 93).

Moreover, we must consider the intrinsic limitations of DTI tractography in cases of excessive peritumoral edema that, in some cases, could seriously hamper the possibility to perform a correct DTI fiber tracking (94, 95), as well as the possible occurrence of brain shift during surgery: the latter is another unavoidable limitation of image-guided surgery, unless intraoperative imaging is employed (96–98). Nevertheless, the use of tailored approaches with minimal brain exposure, and the continuous verification of established superficial anatomical landmarks may reduce inaccuracy due to the brain shift (99).

Finally, we must acknowledge that postsurgical deficits, including VSAs impairment, may be due to other causes than a direct damage during surgery, such as vascular injury to the structures of functional brain networks (100–105). In these cases, the intraoperative visualization of VS network cannot preserve its cortical and/or subcortical structures from indirect ischemic damage caused by surgical damage to vascular structures or postoperative hemodynamic changes. Nevertheless, this aspect is difficult to analyze and is poorly considered in the literature.

However, the present study aims to simply describe a new technique for the advanced visualization of the VS network prior to surgery as well as to report preliminary data about its potential usefulness in the treatment of patients affected by right parietal lobe tumors. It sheds a light on the possibility to use really up-to-date technologies for the non-invasive mapping of brain areas involved in visuospatial processing. This could increase the awareness and the confidence of neurosurgeons with VSAs and VS network, whose importance for patients’ quality of life is still underestimated.

## Conclusions

Cortical mapping using the repetitive nTMS-implemented HVOT is a feasible technique and can be successfully combined with DTI tractography of the SLF branches to achieve a 3D reconstruction of the most important cortico-subcortical components of the brain visuospatial network. Such a reconstruction can be used by neurosurgeons for a customized presurgical planning in patients affected by right parietal lobe tumors with the aim to better assess the risk of surgical damage to the VS network. Moreover, through neuronavigation it could also guide neurosurgeons to identify and preserve the VS network during tumor resection, thus avoiding the occurrence of new subtle postoperative deficits of VSAs. Further larger prospective studies are warranted to confirm our preliminary results.

## Data Availability Statement

The raw data supporting the conclusions of this article will be made available by the authors, without undue reservation.

## Ethics Statement

Ethical review and approval was not required for the study on human participants in accordance with the local legislation and institutional requirements. According to the Italian law, this retrospective observational study was notified to the Comitato Etico Messina. The patients/participants provided their written informed consent to participate in this study.

## Author Contributions

GR: Conception and design of the work, analysis and interpretation of data, drafting the work, revision of the paper for important intellectual content, final approval of the version to be published, agreement to be accountable for all aspects of the work. MQ: Conception and design of the work, interpretation of data, final approval of the version to be published, agreement to be accountable for all aspects of the work. GM: Design of the work, acquisition of data, final approval of the version to be published, agreement to be accountable for all aspects of the work. AC: Design of the work, acquisition of data, final approval of the version to be published, agreement to be accountable for all aspects of the work. VR: Design of the work, acquisition of data, final approval of the version to be published, agreement to be accountable for all aspects of the work. GS: Design of the work, interpretation of data, revision of the paper for important intellectual content, final approval of the version to be published, agreement to be accountable for all aspects of the work. VT: Design of the work, interpretation of data, revision of the paper for important intellectual content, final approval of the version to be published, agreement to be accountable for all aspects of the work. AB: Design of the work, interpretation of data, revision of the paper for important intellectual content, final approval of the version to be published, agreement to be accountable for all aspects of the work. AG: Conception of the work, interpretation of data, revision of the paper for important intellectual content, final approval of the version to be published, agreement to be accountable for all aspects of the work. All authors contributed to the article and approved the submitted version.

## Funding

The study has been funded by the 1) Department of Clinical and Experimental Medicine of the University of Messina, Italy, recipient MQ, and by the 2) European Social Fund (FSE), Call “PON Ricerca e Innovazione 2014-2020 - AIM Attraction and International Mobility”, Activity AIM1839117-3, Line 1, Area SNSI Salute (CUP J44I18000280006), recipients BIOMORF Department of the University of Messina, and GR.

## Conflict of Interest

The authors declare that the research was conducted in the absence of any commercial or financial relationships that could be construed as a potential conflict of interest.

## Abbreviations

RH, Right Hemipsher; RMT, resting motor threshold; HVOT, Hooper visual organization test; KPS, Karnofsky Performance Status; LBT, Line Bisection Task; LH, Left Hemisphere; VS, Visuospatial; WHO, World Health Organization.
